# Tracheal Inflammatory Myofibroblastoma: A Rare Tumor of the Trachea

**DOI:** 10.7759/cureus.4484

**Published:** 2019-04-17

**Authors:** Sonali Gupta, Pradeep Goyal, Yifeng Yang, William Fitzgerald

**Affiliations:** 1 Internal Medicine, St. Vincent's Medical Center, Bridgeport, USA; 2 Radiology, St. Vincent's Medical Center, Bridgeport, USA; 3 Internal Medicine, Frank H. Netter MD School of Medicine, North Haven, USA

**Keywords:** trachea, inflammatory myofibroblastoma, hemoptysis

## Abstract

Inflammatory myofibroblastic tumors (IMTs), also known as inflammatory pseudotumor are rarely reported in the trachea. Although initially considered benign, with the recent evidence of local recurrence after surgical resection, cases of metastatic spread, and advance in cytogenetic analysis, they are now categorized as tumors with malignant potential. Correct histological diagnosis is of utmost importance, as they closely resemble other benign and malignant spindle cell tumors with myofibroblasts predominance. Treatment remains challenging taking into account the location involving the airway. Here, we describe a case of tracheal IMF in an apparently healthy young adult presenting with recurrent hemoptysis.

## Introduction

Inflammatory myofibroblastic tumors (IMTs), also known as inflammatory pseudotumors are rare neoplasms of the respiratory tract. Although they are mostly reported in the lung, tracheal involvement is extremely rare [1]. In the past, they were considered benign; however, with the evidence of local recurrence after surgical resection, cases of metastatic spread, and update on cytogenetic changes, they are categorized as tumors with malignant potential [2]. Correct histological diagnosis is of utmost importance, as they closely resemble other neoplasms such as fibrosarcoma, spindle cell carcinoma or benign conditions such as neurofibroma, pyogenic granuloma, and nodular fasciitis. Treatment remains challenging taking into account the location involving the airway. Here, we describe a case of tracheal IMF in an apparently healthy young adult presenting with recurrent hemoptysis.

## Case presentation

**Figure 1:**
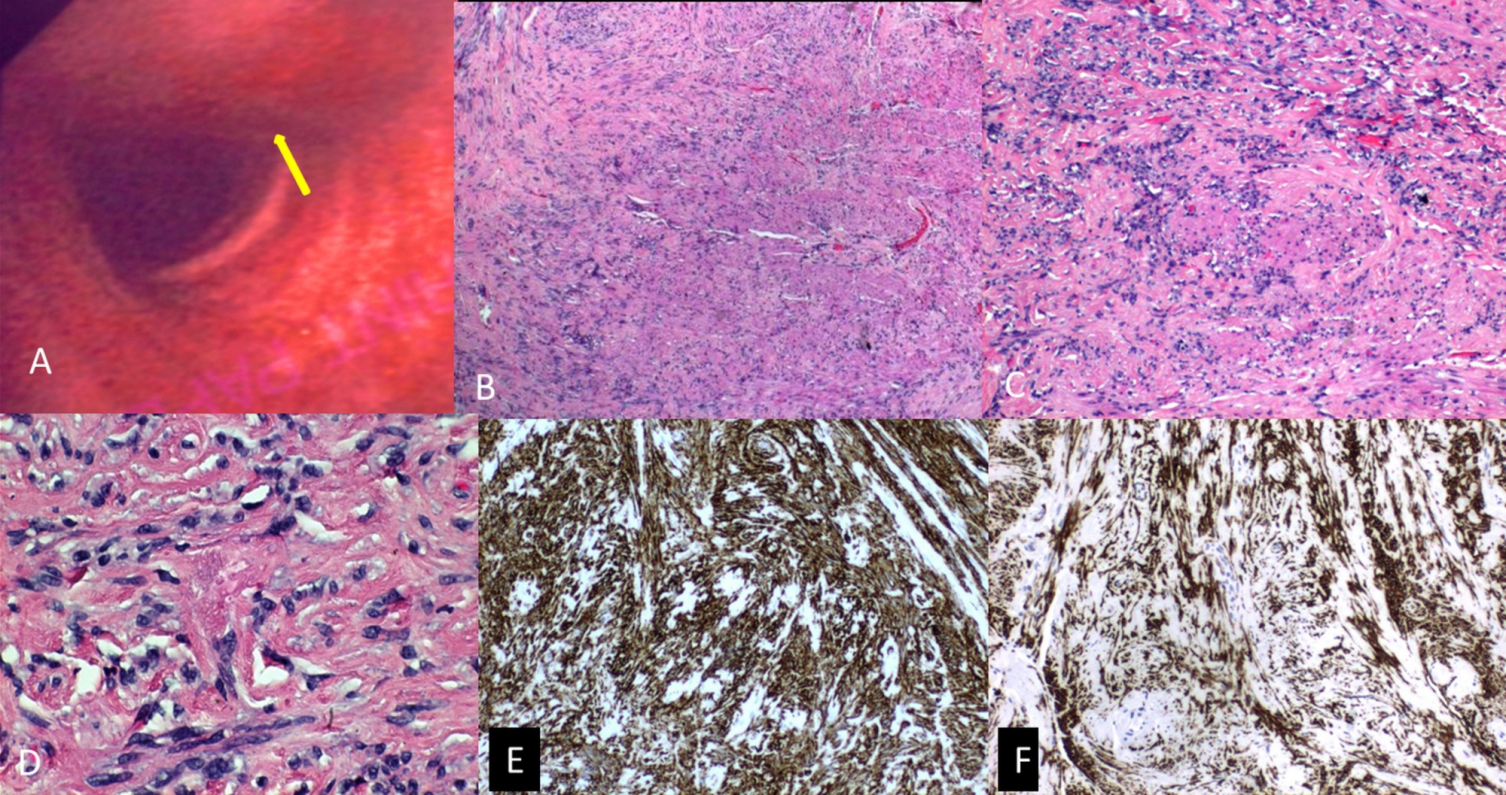
(A) Bronchoscopy image demonstrating a sessile, polypoidal mass (yellow arrow) arising from the left posterolateral aspect of the upper trachea with normal appearing but slightly friable mucosa; (B-D) Histopathology specimens: hematoxylin and eosin stain demonstrating storiform pattern with compact spindle cells surrounded by inflammatory cells in the tumor [(B) 40x; (C) 100x; (D) 400x magnification]; (E) Positive immunohistochemical vimentin in the tumor cells; (F) Positive immunohistochemical smooth muscle actin in the tumor cells

A 34-year-old former smoker presented with recurrent episodes of intermittent hemoptysis for three months. He denied associated dyspnea, chest pain, fever, epistaxis, hematuria, blood in stools or ecchymosis. He was not on any antiplatelet or anticoagulant therapy. He had no significant past medical or family history. Initially hemoptysis appeared to subside after a course of antibiotics suggesting an infective etiology. However, hemoptysis recurred and persisted before presentation. Physical examination including nasopharyngeal examination and vital signs were unremarkable. Laboratory investigations including hemoglobin, platelet count, coagulation profile, erythrocyte sedimentation rate, and other biochemical markers were within normal limits. Chest radiograph and electrocardiogram were unremarkable. Bronchoscopy revealed a sessile, polypoidal mass arising from the left posterolateral aspect of the upper trachea with normal appearing but slightly friable mucosa (Figure 1A). Biopsy of the mass was taken and sent for histology. The remainder of the trachea and bronchi were normal without lesions or excess secretions. Histopathological examination revealed a storiform pattern of compact spindle-shaped cells mixed with inflammatory cells, mostly lymphocytes and plasma cells (Figures 1B-1D). Immunohistochemistry revealed focal positivity for muscle-specific actin, smooth muscle actin, and vimentin (Figures 1E-1F). S-100, cytokeratin, cluster of differentiation (CD) 34, CD 117, and myogenin were negative, compatible with tracheal IMF. The patient was then referred for endoscopic resection.

## Discussion

Tracheal IMF is very rare, accounting for less than 1% of all respiratory tract tumors [1]. It has been reported in the pediatric age group; however, it is rarely seen in the adult population [2-3]. It has been reported throughout the body, with lung parenchyma being the most commonly involved site. However, tracheal involvement in adults is extremely rare with a handful of cases reported in the literature [2-3].

Recent reports suggest an association with recurrent translocations involving 2p23, the anaplastic lymphoma kinase (ALK) gene indicating neoplastic potential [2]. Chromosomal rearrangement involving ALK gene results in the activation of tyrosine kinase receptor, responsible for more aggressive behavior as evident by increased local recurrence after resection and distant metastasis. Trauma, infection, inflammation, and surgery have been reported as potential triggers for the development of IMFs. Recently, IMF was recognized as a low-grade mesenchymal malignancy with an excellent prognosis. Modern immunohistochemistry and fluorescence in situ hybridization can be used to identify the ALK gene, which is reported in up to approximately half of the reported cases and has prognostic significance.

The symptoms are usually nonspecific, and include chronic cough, hemoptysis, pleuritic chest pain, stridor, and post-obstructive pneumonia. Due to its non-specific presentation, it is often missed unless direct laryngoscopy or bronchoscopy is done and the lesion is identified. Grossly, the lesion has been described as nodular, polypoid, or pedunculated [4-6]. They tend to be smooth, fleshy and firm in consistency and the size may range from several millimeters to a few centimeters. To our knowledge, ours is the first reported case of tracheal IMF presenting as recurrent hemoptysis.

Histopathological identification is crucial in diagnosis as it is often misdiagnosed and confused with other neoplasms even by experienced pathologists owing to its low occurrence and a wide variety of presentation. Typically three histologic patterns are recognized [4-6], including spindle or stellate myofibroblasts with abundant inflammatory cells in myxoid stroma or storiform pattern with compact spindle cells surrounded by inflammatory cells or dense collagen with scare cellular pattern. On immunohistochemistry, IMF is typically positive for muscle-specific actin, smooth muscle actin, vimentin, and desmin, while negative for myogenin, cytokeratin, and S100. Differentials include benign and malignant spindle cell tumors with myofibroblasts predominance.

It is commonly managed conservatively with endoscopic laser photocoagulation, cryoablation, or electorcautery [2,4]. However, 18%-40% recurrence rate has been reported after conservative management. A few reports suggest radical treatment involving surgical resection of the involved tracheal rings in cases of transmural involvement or radiotherapy for surgically unfit patients [7-8]. Although the prognosis after radical resection is excellent, the procedure is associated with higher morbidity and complication rate. Cerfolio reported a five and 10-year survival rate of 91% and 77% respectively, after radical resection of IMFs involvement lung [9]. Fabre et al. also reported an excellent 10-year survival rate after surgical resection [10]. However, the data for tracheal IMFs is limited.

Newer therapies including inhibitors of the tyrosine kinase domain of ALK protein and crizotinib show promising results especially in patients with ALK-translocated IMT [11]. It can be used initially for unresectable tumors to facilitate complete surgical removal or combined with surgery in cases complicated with local recurrence.

## Conclusions

IMF should always be considered in the differential diagnosis for young adults presenting with recurrent hemoptysis. Due to the rarity and scarce literature pertaining to its occurrence, it is often misdiagnosed. It is important for clinicians to be well aware of this entity and to initially consider lesser invasive treatment options including endoscopic resection for endo-luminal lesions, which offers excellent results.
